# Evolution of micro-pores in Ni–Cr alloys via molten salt dealloying

**DOI:** 10.1038/s41598-022-20286-5

**Published:** 2022-12-01

**Authors:** Lin-Chieh Yu, Charles Clark, Xiaoyang Liu, Arthur Ronne, Bobby Layne, Phillip Halstenberg, Fernando Camino, Dmytro Nykypanchuk, Hui Zhong, Mingyuan Ge, Wah-Keat Lee, Sanjit Ghose, Sheng Dai, Xianghui Xiao, James F. Wishart, Yu-chen Karen Chen-Wiegart

**Affiliations:** 1 Department of Materials Science and Chemical Engineering, Stony Brook University, Stony Brook, NY USA; 2 Department of Chemistry, Stony Brook University, Stony Brook, NY USA; 3 Chemistry Division, Brookhaven National Laboratory, Upton, NY USA; 4 Department of Chemistry, University of Tennessee, Knoxville, TN USA; 5 Chemical Sciences Division, Oak Ridge National Laboratory, Oak Ridge, TN USA; 6 Center for Functional Nanomaterials, Brookhaven National Laboratory, Upton, NY USA; 7 National Synchrotron Light Source II (NSLS-II), Brookhaven National Laboratory, Upton, NY USA; 8 Joint Photon Sciences Institute, Stony Brook University, Stony Brook, NY USA

**Keywords:** Materials science, Materials for energy and catalysis, Porous materials

## Abstract

Porous materials with high specific surface area, high porosity, and high electrical conductivity are promising materials for functional applications, including catalysis, sensing, and energy storage. Molten salt dealloying was recently demonstrated in microwires as an alternative method to fabricate porous structures. The method takes advantage of the selective dissolution process introduced by impurities often observed in molten salt corrosion. This work further investigates molten salt dealloying in bulk Ni–20Cr alloy in both KCl–MgCl_2_ and KCl–NaCl salts at 700 ℃, using scanning electron microscopy, energy dispersive spectroscopy, and X-ray diffraction (XRD), as well as synchrotron X-ray nano-tomography. Micro-sized pores with irregular shapes and sizes ranging from sub-micron to several microns and ligaments formed during the process, while the molten salt dealloying was found to progress several microns into the bulk materials within 1–16 h, a relatively short reaction time, enhancing the practicality of using the method for synthesis. The ligament size increased from ~ 0.7 μm to ~ 1.3 μm in KCl–MgCl_2_ from 1 to 16 h due to coarsening, while remaining ~ 0.4 μm in KCl–NaCl during 16 h of exposure. The XRD analysis shows that the corrosion occurred primarily near the surface of the bulk sample, and Cr_2_O_3_ was identified as a corrosion product when the reaction was conducted in an air environment (controlled amount sealed in capillaries); thus surface oxides are likely to slow the morphological coarsening rate by hindering the surface diffusion in the dealloyed structure. 3D-connected pores and grain boundary corrosion were visualized by synchrotron X-ray nano-tomography. This study provides insights into the morphological and chemical evolution of molten salt dealloying in bulk materials, with a connection to molten salt corrosion concerns in the design of next-generation nuclear and solar energy power plants.

## Introduction

Nanoporous metals have attracted significant attention due to their high specific surface area, tunable pore size, low density, high structural stability, and high electrical conductivity for various applications such as catalysts^1^, sensors^2^, and energy storage materials^3–5^. Dealloying is one of the methods used to fabricate three-dimensional (3D) micro/nanoporous materials. By definition, dealloying is a materials process that selectively removes one or more compounds from a parent alloy, leaving the remaining element(s) to rearrange and form a porous structure^6^. Porous materials created by dealloying methods show high efficiency for catalysis and energy storage due to large, electrochemically-active surface area in the network architecture^7^.

Conventionally, aqueous solution dealloying (ASD) has been a well-studied process that involves free corrosion or selective leaching from alloys^8^. However, corrosive acids and bases that are commonly used to remove the less noble elements, such as HNO_3_ and NaOH solutions, produce hazardous wastes during the dealloying process^9–11^. Moreover, ASD is limited to fabricate porous noble metals such as Au^9^, Pt^10^, and Pd^12^. A range of different pore sizes in the nm regime and hierarchical designs have been achieved, such as a ultrafine nanoporous metal by low-temperature dealloying (~ 5 nm pore size)^13^, an ultrafine spongy morphology that contains polygonal pores (~ 12 nm)^12^, and a hierarchical bicontinuous nanoporous structure^10^. Generally, ASD is suitable to fabricate ultrafine nano-sized 3D bicontinuous porous materials at the nm and tens of nm length scales. Liquid metal dealloying (LMD) can fabricate nanoporous structures through solubility differences between alloy components and a metallic melt^14^. Due to the relatively higher reaction temperature compared to ASD, the size of the porous structure is generally larger via LMD. With this method, 3D connected open-cell porous Ti of ~ 200 nm^14,15^, truncated cube, spherical polygon and rod-like Fe^16^, and nanoporous high-entropy alloys^17^ were fabricated in previous studies. However, an acid solution and chemical etching are required to remove the residual metal from the bicontinuous composites to form a porous structure^14^. Using a metal in the solid-state to drive dealloying processes, known as solid-state interfacial dealloying (SSID) or solid-state metal dealloying (SSMD), has also been demonstrated in bulk materials^18^ and thin film forms^17,19^. In recent research, a vapor-phase dealloying (VPD) method was developed to selectively evaporate a component from an alloy in high-vacuum by utilizing the vapor pressure differences between the constituent components^20–25^. The pore size and porosity can be tuned by changing the VPD temperature, time, and composition of the precursor. Overall, the dealloying methods provide a suite of versatile tools to fabricate 3D bicontinuous open porous metals and metallic composites, with pore sizes ranging from nm to tens of nm and to microns.

As an alternative approach, molten salt has been demonstrated as a promising dealloying agent to fabricate porous structures^26,27^. Molten salt dealloying (MSD) was motivated by the salt-induced corrosive attack of metals and alloys in next-generation nuclear reactor system^28^, concentrated solar power plants^29–31^, and waste incineration plants^32,33^. Molten salt corrosion is primarily driven by impurities, including water, oxygen, and metal ion contaminants introduced from the environment or structural materials^28,31,34^. The phenomenon has been widely studied in the context of better understanding corrosion mechanisms to prevent material degradation^28^.

According to their redox potentials, Cr can be corroded preferentially from an alloy into the molten salt compared with Ni, indicating that Ni–Cr alloy would be a suitable binary alloy for molten salt dealloying^28,35^. Pore formation in Ni-based structural alloys has been observed in studying microstructural evolution in the context of molten salt corrosion studies^36,37^, but the emphasis has not been chiefly on dealloying or nanoporous material fabrication. Chloride salts, such as NaCl, KCl, and MgCl_2_, are abundant materials and have the benefits of relatively low cost, wide operation temperatures, and excellent heat transfer properties^38^. Previous research has investigated the corrosion behavior of various alloys in chloride salt systems. Binary and ternary chloride salt mixtures such as KCl–MgCl_2_ and NaCl–KCl–ZnCl_2_ have been used to lower melting points and operational temperatures. The corrosion behavior and resistance of a series of Inconel alloys and other Ni-based alloys has been studied in several salt systems^36,37,39^. A pervasive porous network was discovered in Inconel 601 after 120 h corrosion in molten NaCl-Na_2_SO_4_ salt^36^. To fundamentally understand molten salt dealloying and corrosion phenomena, in situ synchrotron X-ray nano-tomography was applied to a Ni–20Cr micro-wire in the KCl–MgCl_2_ system, providing insights into kinetic evolution of Cr leaching with bi-continuous structural formation^27,40^. Long-range diffusion was determined to be the rate-limiting mechanism in the MSD, while coarsening dominated the morphological evolution of long-term corrosion. Although the kinetics concerning the dealloying and coarsening mechanisms were investigated in the previous study, it was conducted in a microwire geometry and limited to a single salt composition under an inert environment. Understanding the MSD process in planar, bulk sample systems with a wider range of salts and in an atmospheric environment is needed to develop MSD methods to fabricate bicontinuous metals with tunable size and porosity for functional applications. The understanding of these processes will also benefit the communities concerned with MSD as a detrimental phenomenon leading to materials corrosion.

In this research, we studied MSD in bulk Ni–20Cr foils to form porous structures. During the process, Ni–20Cr foil was corroded by molten KCl–MgCl_2_ in vacuum as well as KCl–NaCl salt mixtures in vacuum and in air at 700 °C. 700 ℃ was chosen to be the operating temperature in this was because the melting temperature is 664 ℃ for KCl–NaCl (~ 50–50 mol%) and 430 ℃ for eutectic KCl–MgCl_2_^41,42^. Without using hazardous dealloying agents, the salt residue can be easily washed away by water. The morphological and chemical evolution was characterized using scanning electron microscopy (SEM), energy dispersive spectroscopy (EDX), and X-ray diffraction (XRD), as well as synchrotron X-ray nano-tomography. The shape and size of the features were determined by percolation dealloying, coarsening and oxidation reactions. Overall, the work using Ni–20Cr foil and binary salt systems provided insights into the feasibility of dealloying bulk alloys in molten salt. This work also furthered our understanding on the influence of the salt system and atmosphere in dealloying the bulk material. In addition to expanding the molten salt dealloying method to a wider range of alloys, salts and treatment conditions, future fundamental research can build upon these findings to further reveal the underlying chemical and electrochemical reactions and materials’ morphological evolution kinetics in molten salt dealloying.

## Experimental methods and analysis

Equimolar (1:1) KCl–MgCl_2_ and KCl–NaCl salt mixtures were selected as the dealloying agent. The MgCl_2_ salt was purified using fractional distillation from commercial anhydrous salt and titrated to determine the oxide content. Details of the purification process can be found in prior publications^27^. The purified MgCl_2_, KCl (Sigma Aldrich, 99.999% trace metals basis, -10 mesh) and NaCl (Sigma Aldrich, ≥ 99.0% purity, powder) were ground and mixed with a mortar and pestle in a 1:1 molar ratio inside of a glovebox.

As-rolled, 100 µm-thick Ni–20Cr (Cr 20 wt%) foil (Goodfellow, USA-NI050235) was cut into 2 cm × 2 mm strips. Prior to the corrosion experiments, the Ni–20Cr foil samples were cleaned and sonicated by isopropanol, ethanol, and deionized (DI) water in sequence. Quartz capillaries (2.0 mm diameter, Charles Supper, 20-QZ) were baked out at 500 °C for 45–60 min to remove any moisture or potential organic species adsorbed on the surface.

Three types of samples were prepared: Ni–20Cr alloy with KCl–MgCl_2_ in a capillary sealed under vacuum, and the same alloy with KCl–NaCl in both vacuum and in air-filled sealed capillaries. Throughout this report any future references to ‘in air’ dealloying refers to dealloying within the air-filled sealed capillary, not in ambient environment. Inside an Ar-filled glovebox, one piece of Ni–20Cr foil was inserted into one quartz capillary for each of the reaction conditions and reaction times. A powder of the desired salt mixture (~ 0.3 g) was then poured into the quartz capillary. The filled capillary was then connected to a vacuum adapter built in-house and transferred out of the glovebox; a quarter-turn instrument plug valve (Swagelok, SS-2P4T4) was closed prior to the transfer to ensure that the sample was not in contact with air as shown in Fig. S1a. For samples prepared in vacuum, the vacuum adapter with the filled capillary was pumped by a roughing pump for 10 min. For samples prepared in air-filled capillaries, the vacuum adapter was open for 10 min to allow the air to fill the capillary. Both types of capillaries were then flame-sealed using a miniature benchtop hydrogen torch (Rio Grande, model L45) and mounted onto a stainless-steel sample holder designed for capillaries shown in Fig. S1b. The samples were then transferred into a box furnace at 700 °C and heated for various designated times: 1, 2, 4, and 16 h. After the corrosion experiments, the corroded foil samples were removed from the furnace. The capillaries were then broken, and the samples were sonicated in DI water for 20 min to remove residual salts. The corrosion conditions for samples used in this study with different heating times are summarized in Table 1.

**Table 1:** A summary of experimental conditions for samples used in this study.

Salt mixture (All 50:50 mol. %)	Atmosphere	Heating temperature (°C)	Heating time (h)
KCl–MgCl_2_	Vacuum	700	1
KCl–NaCl			2
			4
KCl–NaCl	Air		16

In order to characterize the cross-sectional morphology of the corroded samples, a cross-sectional specimen was prepared by Waldvogel Metallurgical, *Inc.* The corroded foil samples were repositioned on the top side of an aluminum disk and vacuum mounted in a metallographic grade epoxy and cured for 12 h at room temperature. The metallographic sectioning was performed with standard grinding and polishing techniques using silicon carbide paper, diamond polishing compounds, alumina suspensions, and colloidal silica suspensions following the specification ASTM E3 “Standard Methods of Preparation of Metallographic Specimens” and ASTM B487 “Standard Test Method for Measurement of Metal and Oxide Coating Thickness by Microscopical Examination of a Cross Section” ^43–45^. The cross-sectional specimen was coated by Au (50 nm thickness measured by the calibrated quartz crystal thickness monitor in the desktop sputter coater (Denton Vacuum, DESK V) for SEM analysis) to improve conductivity prior to imaging.

Scanning Electron Microscopy (SEM, JEOL JSM-7600F) was used to obtain surface and cross-sectional SEM images. The ligament width of all samples was measured from the SEM images with the surface view; 20 measurements were taken using Fiji, an ImageJ package^46^, and averaged. The thickness of the sample was measured from cross-sectional SEM images. Three measurements were taken by the built-in distance measurement tool in SEM and averaged (Fig. S2). Energy Dispersive Spectroscopy (EDX) analysis was conducted to study the elemental distribution within the samples. The SEM and EDX analyses were performed at the Center for Functional Nanomaterials (CFN) of Brookhaven National Laboratory (BNL).

The ex situ X-ray diffraction experiment was performed at the X-ray Powder Diffraction (XPD, 28-ID-2) beamline at National Synchrotron Light Source II (NSLS-II), Brookhaven National Laboratory (BNL). The experiment was conducted with a photon beam energy of ~ 67.1 keV (X-ray wavelength at 0.1846 Å) and a nominal beam size of 0.5 mm × 0.5 mm. An X-ray detector with 2048 × 2048 pixels was used to obtain the diffraction signals, with the size of each pixel being 200 × 200 µm^2^. Each XRD pattern was calibrated with a Ni standard using the python-based software Dioptas^47^. The X-ray diffraction analysis in a reflection geometry was also conducted for a surface-sensitive measurement by Rigaku SmartLab at CFN, BNL. 2-theta scans were collected using a 1D detector with a Cu-K_α_ radiation (0.04 degrees step size, scan rate: 1.6 deg/min). The angle of incidence was fixed at 5 degrees. The X-ray fluorescence signals from the samples were filtered out by the built-in energy discrimination function in the detector to reduce the background. The phase identification was then conducted using Jade (Materials Data, Inc.) and PDF-4+ 2021 commercial software packages.

To further characterize the 3D structure resulting from the corrosion reaction, the Ni–20Cr sample corroded in KCl–NaCl in air for 16 h was characterized by synchrotron X-ray nano-tomography. A micro-pillar sample for nano-tomography was prepared by focused ion beam milling and lift-out at CFN, BNL. The sample was prepared following an established procedure that was detailed in a prior publication^48^. The final cylinder was 9–10 µm in diameter and ~ 28 µm in height (Fig. S3).

X-ray nano-tomography measurements were conducted at the Full-Field X-ray Imaging (FXI, 18-ID) beamline at NSLS-II, BNL. The incident X-ray energy used 8.4 keV, just above the Ni K-edge of 8.333 keV. The sample was measured in a fly-scan mode with a rotation range of 200°, an exposure time of 100 ms, and a rotation speed of 2°/s. The pixel size for data collection is 20.09 nm which was determined by the magnification chosen for the microscopy (~ 324) and the pixel size of the lens-coupled charge-coupled device (CCD) detector (6.5 μm), The data was then further binned with 2 × 2 pixels during the tomographic reconstruction, and hence the reconstructed volumes have a pixel size of 40.18 nm. A total of 909 projection images were collected. The nano-tomography data was reconstructed with pixel size of 40.18 nm (2 × 2 binned) using Tomopy^49^. Visualization of 2D pseudo cross-section images and 3D volumes was conducted in commercial software Avizo (Thermo Fisher Scientific, v. 9.3) on the reconstructed data.

## Results and discussion

### Surface morphological and chemical evolution

**Figure 1:**
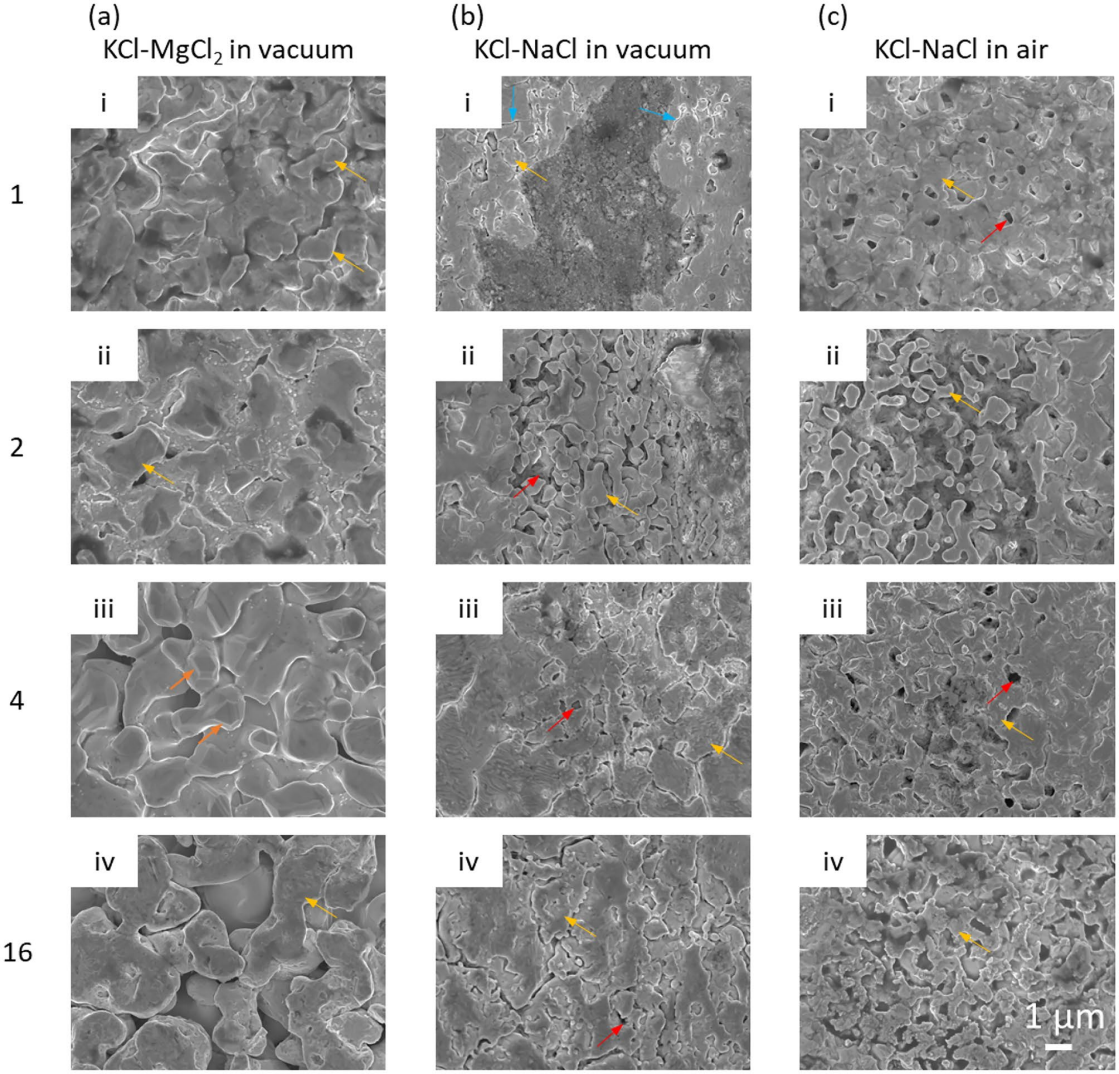
SEM images showing the surface morphology of Ni–20Cr alloy corrosion in molten salts under different conditions: (**a**) KCl–MgCl_2_ in vacuum, (**b**) KCl–NaCl in vacuum, and (**c**) KCl–NaCl in air for (i) 1 h, (ii) 2 h, (iii) 4 h, and (iv) 16 h. The orange arrows indicate the facets formed on the surface after corrosion. The blue arrows indicate the cracks, the red arrows show the pores, and the yellow arrows refer to the ligaments.

**Figure 2:**
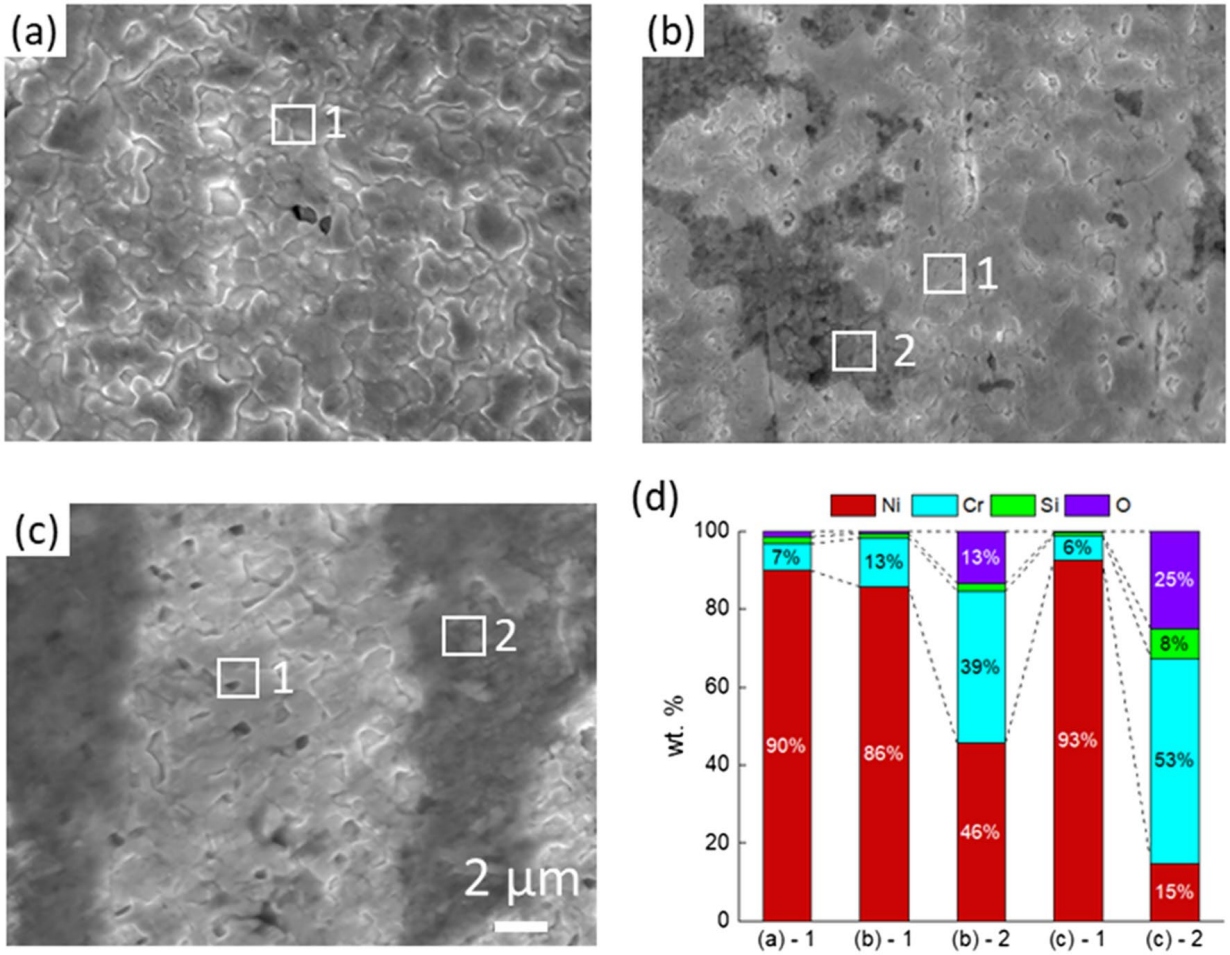
SEM images and elemental analysis of Ni–20Cr alloy corrosion in molten salts under different conditions for 1 h: (**a**) KCl–MgCl_2_ in vacuum, (**b**) KCl–NaCl in vacuum, (**c**) KCl–NaCl in air, and (**d**) the elemental analysis by EDX.

Figure 1 shows the surface SEM images of the morphological changes of the Ni–20Cr samples corroded under the three different conditions for 1, 2, 4, and 16 h. Large pores and ligaments were observed on the surface of Ni–20Cr foils after corrosion in KCl–MgCl_2_ in vacuum as shown in Fig. 1a. With increasing corrosion time, the porous structure continues to grow, indicating that the salt not only corrodes the surface of foil but also progresses into the materials. In Fig. 1a-iii, facets could be identified on the ligaments. Coarsening also occurred, which led to smoothening the surface as shown in Fig. 1a-iv. In Fig. 2, the EDX analysis confirmed that Cr dissolution was the main reaction in this molten-salt corrosion process, effectively a dealloying reaction of Ni–20Cr. Approximately 7.0 wt% of Cr remains as residual on the surface after corrosion for 1 h.

Figure 1b shows the corrosion of the materials in KCl–NaCl treated in vacuum. The corrosion occured at the cracks, forming small pores and particles. The cracks became more dense and continuous after prolonged corrosion time. In Fig. 2b, two different regions were idenified based on the EDX analysis: a Ni-rich region and an oxide region. The corrosion in the Ni-rich region is relatively mild since a significant amount of the Cr content remains in the system, which only reduced from 20 to 13 wt%. The oxide region contains relatively high amounts of Cr and O. Since the materials were treated in vacuum, a small amount of trace oxygen may come from the salt. Figure 2c displays the evolution of the Ni–20Cr alloy from the corrosion in KCl–NaCl in air. The corrosion is more rapid due to the presence of moisture and oxygen in the air. Micropores formed within just one hour of exposure. As exposure time increased, the edges of the features formed during corrosion became sharper, which was different from the corrosion behavior in KCl–MgCl_2_ in vacuum where coarsening drove the growth of the ligaments and pores, commonly observed in other porous metals created by dealloying^50^. As shown in Fig. 2c, the Ni-rich regions and the oxide regions were analyzed. The Cr content was lower than 10 wt% in the Ni-rich region. For the oxide region, the Cr signal became dominant while the Si content also increased (~ 7 wt%). The higher Si content in the samples corroded in air indicated that the molten salt may also react with the quartz capillary under the ambient environment.

### Cross-sectional analysis of the morphological evolution

**Figure 3:**
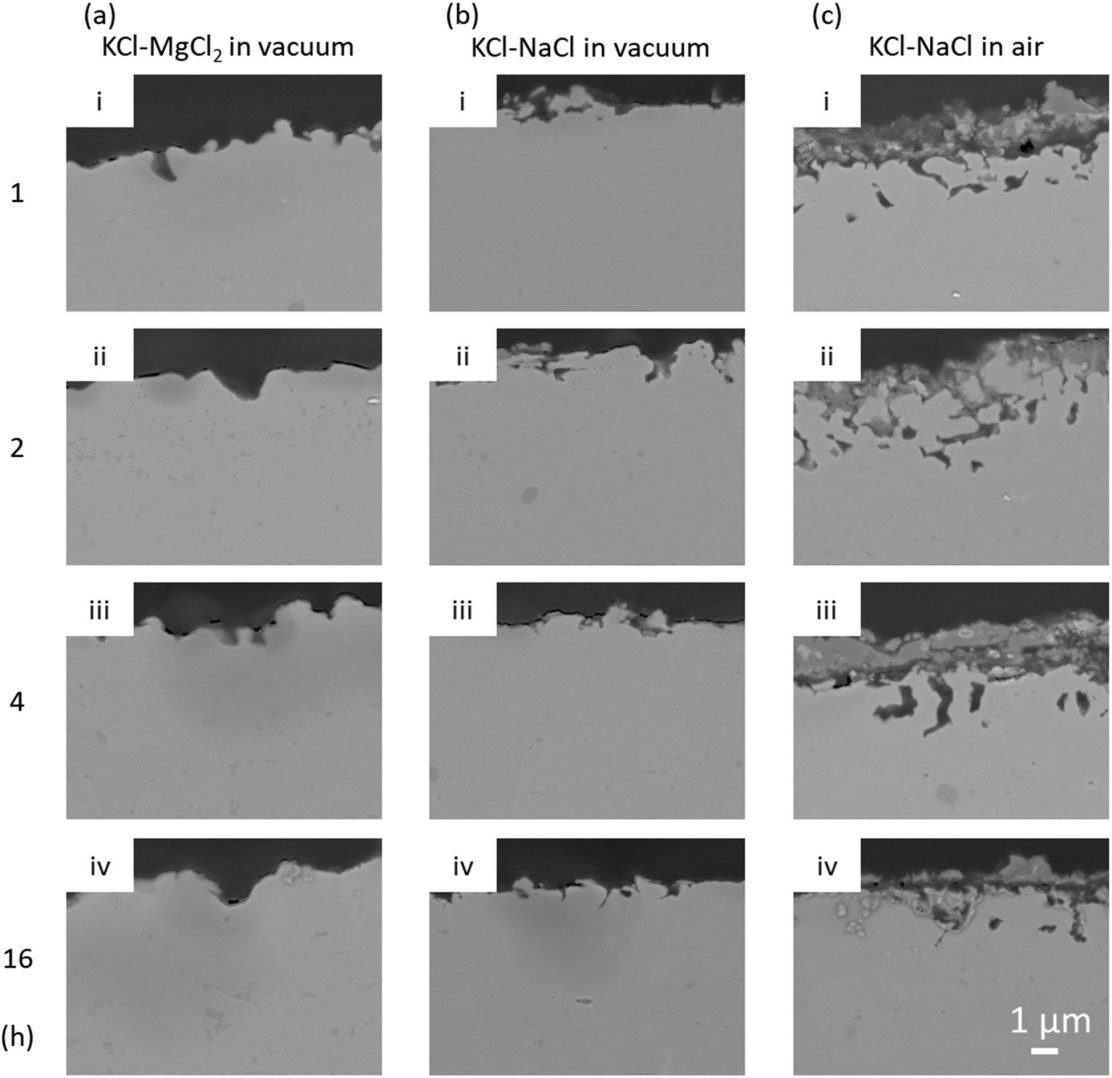
SEM images showing the cross-section view of Ni–20Cr corrosion in molten salts under different conditions: (**a**) KCl–MgCl_2_ in vacuum, (**b**) KCl–NaCl in vacuum, and (**c**) KCl–NaCl in air for (i) 1 h, (ii) 2 h, (iii) 4 h, and (iv) 16 h.

**Figure 4:**
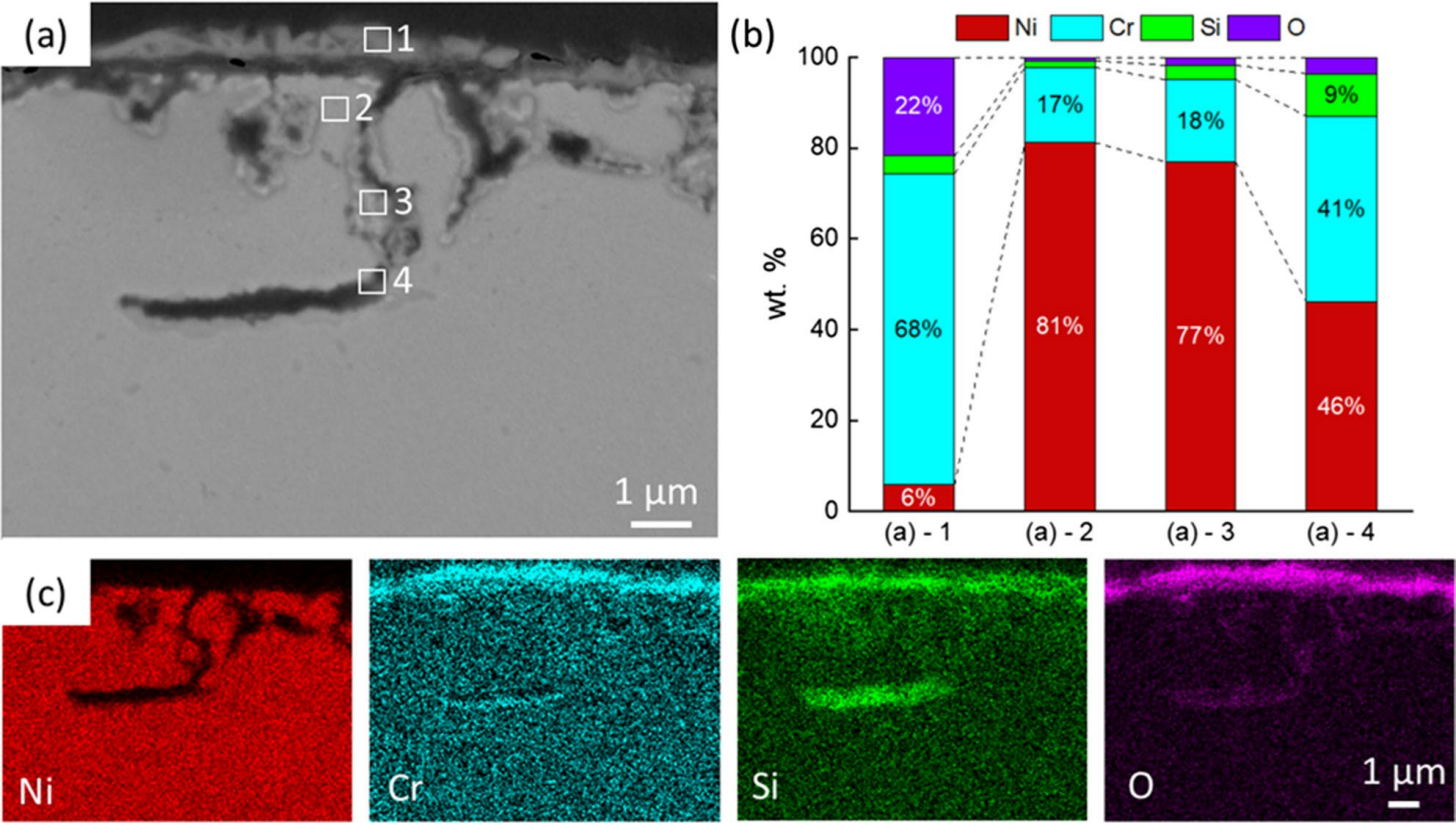
Morphological and elemental analysis of Ni–20Cr corrosion in KCl–NaCl in air for 16 h: (**a**) Cross-sectional SEM image, (**b**) elemental analysis by EDS, (**c**) element mapping by EDX showing the elemental distribution.

The cross-sectional SEM images of corroded samples are shown in Fig. 3. It can be seen that the corrosion did not progress significantly into the bulk in both the KCl–MgCl_2_ and KCl–NaCl in vacuum, with a corrosion depth only ~ 1.8 and ~ 1.4 µm after 16 h of reactions, respectively. In contrast, when Ni–20Cr foil was corroded in molten KCl–NaCl in air, the moisture and oxygen in the system will drive the corrosion, forming a relatively thick Cr_2_O_3_ layer (~ 2.5 µm). The corrosion depth of ~ 2.5 µm was also further into the bulk materials compared with the other two conditions conducted under vacuum. As shown in Fig. 4, the EDX analysis shows that the Cr and O contents are higher in the region near the surface. Chromium and O_2_ from the air react to form the Cr_2_O_3_ layer. However, the EDX mapping shows that the corrosion cannot progress to the deeper region and oxygen is only distributed on the surface and in pores. The corrosion may be limited by the oxidation with relatively lower oxygen in the environment. The amount of Cr in the foil is approximately 100 times higher than O_2_ in the air-filled capillary. However, other reaction mechanisms may also need to be considered. For instance, literature has shown that molten salt corrosion can continue to progress in an inert environment. Further analysis should consider the competition and balance between the mass transport and reaction kinetics.

### Feature size analysis and thickness analysis

**Figure 5:**
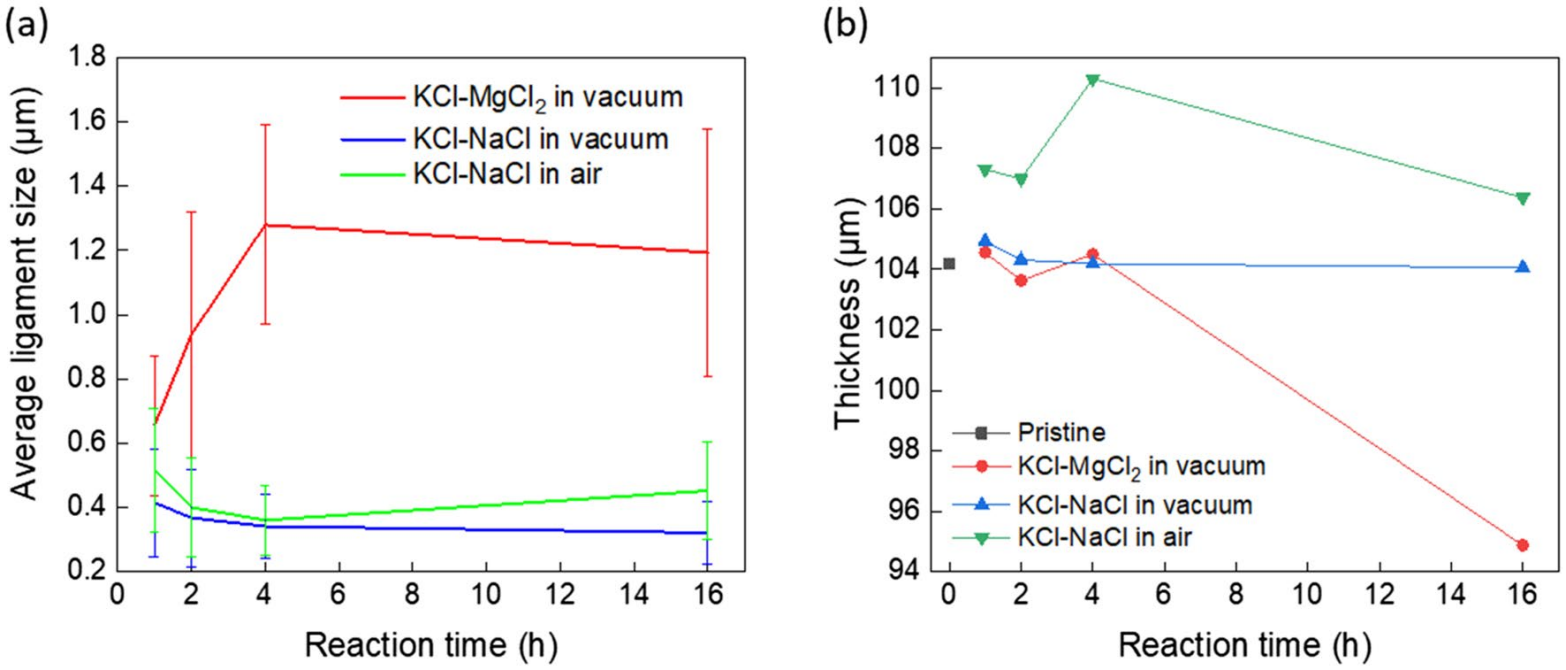
(**a**) Average ligament width in Ni–20Cr foils after corrosion in different molten salt conditions as a function of reaction time at 700 °C. The values for standard deviation of the measurements are also labeled as error bars. (**b**) Thickness of Ni–20Cr foils versus reaction time after corrosion in different salt conditions as a function of reaction time at 700 °C.

The average ligament width with the standard deviation was quantitatively analyzed by SEM images (20 measurements in each image) as shown in Fig. 5a. The feature size increased significantly up to 1.28 µm from 0.66 µm after corrosion in KCl–MgCl_2_ in vacuum. The full analysis is shown in Fig. S4 (Supporting Information). The increasing feature size here primarily corresponds to a coarsening process, commonly observed in porous metallic structures formed by dealloying^51,52^. In contrast, the corrosion in KCl–NaCl in both vacuum and air formed smaller ligament sizes that did not change during the 16 h of exposure. The slower morphological coarsening may be attributed to the presence of the oxides, primarily due to the use of a less pure NaCl starting material where a higher water and oxygen contents may be present. It has been shown in prior studies that surface oxides or doped elements can create defects, thereby acting as diffusion barriers to slow down or even prohibit the coarsening of bicontinuous structures^52–55^. The process may benefit certain functional applications where a smaller ligament size and thus higher surface areas are desirable to maintain a higher surface reactivity.

The foil thickness versus reaction time was measured in the cross-sectional SEM micrographs shown in Fig. 5b. During corrosion in KCl–MgCl_2_ salt, the thickness of the Ni–20Cr foil did not change significantly in the beginning but decreased to ~ 94.88 µm after 16 h of corrosion, likely due to the loss of material. However, the corrosion in KCl–NaCl in vacuum did not alter the thickness of the foils significantly. The variation of the thickness throughout the 16 h reaction is < 1.0%, and the thickness remains at 104.06 µm, similar to the initial thickness of 104.57 µm, since most impurities are removed from the vacuum system. It can be seen that the thickness of the Ni–20Cr foil after 1 h corrosion in KCl–NaCl in air increases significantly to 107.31 µm, likely due to the newly-formed Cr_2_O_3_ oxide layer.

### X-ray diffraction characterization

**Figure 6:**
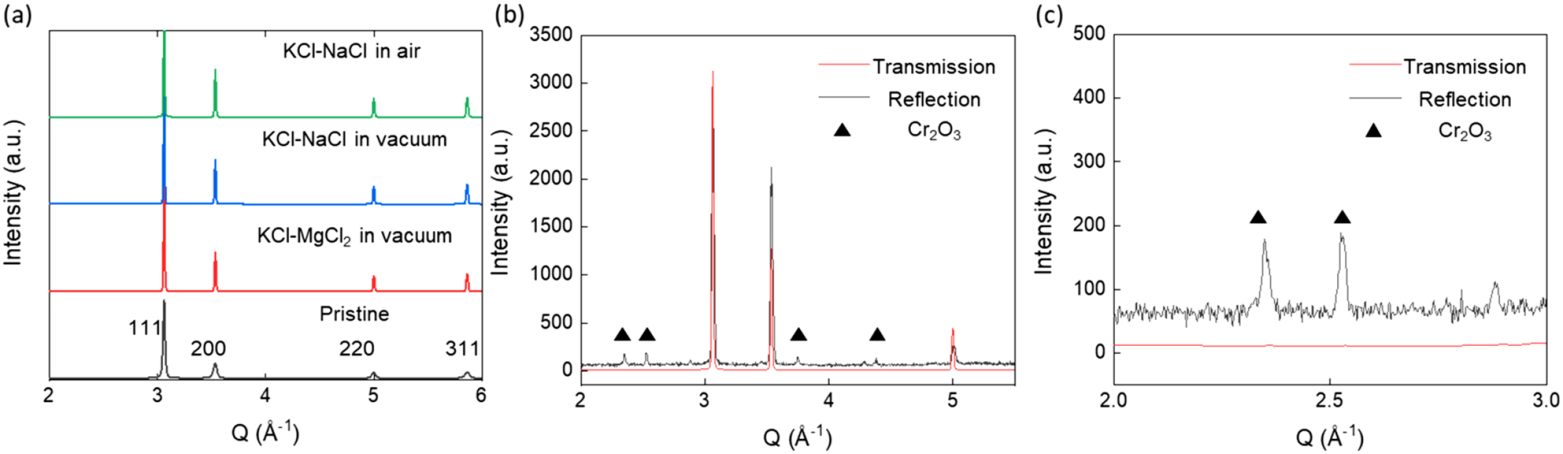
(**a**) XRD patterns of Ni–20Cr foils before and after corrosion in different molten salt conditions for 1 h at 700 °C. The peaks correspond to Ni–20Cr. (**b**) Comparison of XRD pattern of Ni–20Cr foils after corrosion in KCl–NaCl in air, with transmission versus reflection geometries. The main peaks correspond to Ni–20Cr, with Cr_2_O_3_ peaks labeled with triangles. A zoom-in view of (**b**) is shown in (**c**) to focus on the Cr_2_O_3_ peaks. The x-axis was converted to the Q-space for comparing the signals measured with different incident X-ray energies in the laboratory and synchrotron instrument.

Figure 6A shows the XRD analysis results of Ni–20Cr foil corroded in the molten KCl–MgCl_2_ and KCl–NaCl salt mixtures in vacuum and air. The positions of characteristic peaks, (111), (200), (220), (311), remain unchanged after corrosion, indicating the bulk of the sample remains mostly as Ni–20Cr, or only partially becomes Ni-rich from the Cr leaching. Here, the XRD measurement in transmission geometry is not sensitive enough to detect the corrosion products on the surface. A zoom-in view of the XRD analysis is shown in Fig. 6c, and no Cr_2_O_3_ peaks were identified in the transmission geometry. Using the XRD analysis in the reflection geometry, the presence of the Cr_2_O_3_ phase (JCPDF Card No.:01-073-4336) was confirmed for the sample reacted in air. In Fig. 6b, the Cr_2_O_3_ peaks were identified in the diffraction pattern with diffraction peaks at Q values of 2.357 Å^−1^ (104), 2.533 Å^−1^ (110), 3.757 Å^−1^ (116), and 4.388 Å^−1^ (300). The formation of Cr_2_O_3_ was confirmed within the oxide layer in cross-sectional observation. In aqueous hot corrosion studies of steel in a saturated solution of NaCl, it was shown that Cr and Cr_2_O_3_ could react with NaCl in the presence of oxygen to form Na_2_CrO_4_ (Cr^6+^), a carcinogenic compound as with other Cr (VI) compounds^56^. Here we only identified Cr_2_O_3_ as the reaction product. The Na_2_CrO_4_ formation may be inhibited due to the low amount of O_2_ in the environment, which could play a key role in impacting the surface-mediated processes^57^ including corrosion as shown in a recent multimodal study^58^ but also other functional properties such as catalytic reactivities. The effect of a molten salt environment in contrast to the aqueous corrosion environment should also be discussed further. Note that Cr_2_O_3_ has been found to play a key role in other corrosion studies.

With the oxygen in the environment, the corrosion was enhanced, and chromium oxides formed. The newly-formed oxides may be beneficial or disadvantageous for the porous materials depending on the application. On one hand, the oxides can function as catalysts. The Cr_2_O_3_ nanoparticles are non-noble metal catalysts for CO oxidation with high performance at low temperature^59^. The oxides can also be assembled into composites such as Ni/NiO–Cr_2_O_3_ composite, which was designed as an electrocatalyst for hydrogen evolution reaction (HER) in alkaline electrolyte^60^. During the HER cycle, the Cr_2_O_3_ component can stabilize the NiO_x_ component and maintain the catalyst’s activity^60,61^. However, the oxide layer may also hinder the catalyst efficiency for porous metal and nanoparticles. In previous research on the oxidation of nanoparticles, it was found that the oxidation will affect the particle shape, activation energy and hinder the active site^61–64^. Oxidation is undesired and destructive for catalysis if the goal is to fabricate catalysts with pure porous metals. Overall, it is critical to consider the presence of the oxides on the surface when designing porous metals through the molten salt dealloying method in an air-filled environment.

### 3D morphological analysis by X-ray nano-tomography

**Figure 7:**
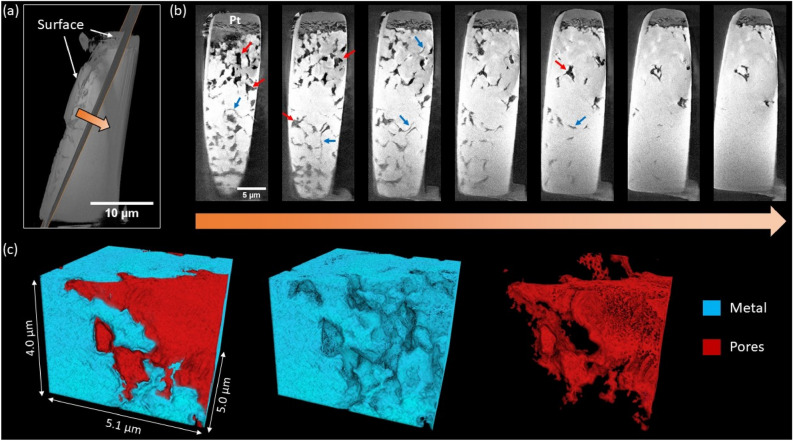
Synchrotron X-ray nano-tomography on Ni–20Cr corrosion in KCl–NaCl in air for 16 h: (**a**) Diagonal slice of interest used for depth profiling. (**b**) Pseudo-2D cross sections along depth direction, spaced approximately 0.4 μm apart. Some larger pores and intergranular cracks are indicated using red and blue arrows, respectively. (**c**) 3D volume rendering of near-surface region displaying pore formation.

The Ni–20Cr foil corroded in KCl–NaCl in air for 16 h at 700 °C was characterized by X-ray nano-tomography to observe the 3D morphology of the porous structure (Fig. 7). The surface morphology was preserved by a Pt protection layer. The surface became rough after corrosion. Figure 7b shows the 2D cross-sectional images along with the depth direction from the side surface. Beneath the surface significant pore formation was observed, especially near the surface of the foil where corrosion was severe, showing both cracks and large pores. In contrast, in the deeper region, there was less corrosion with mainly grain boundary cracks as also seen in the literature of molten salt corrosion^28,65,66^. It is known that atoms within the grain boundary have fewer nearest neighbors on average than the atoms within the grains, and therefore are more susceptible to corrosion atacks^8^. When molten salts react with the Ni–20Cr, Cr atoms within the grain boundaries would be preferentially dissolved first, leaving intergranular cracks. The larger pores suggest that the corrosion has propagated into the grains with longer heating time. Additionally, from both the pseudo cross-sectional images and 3D morphological analysis (Fig. 7c and Supplementary Movie 1), the grain boundary cracks were not yet present throughout the whole sample but mostly connected, with the deeper region >  ~ 3 μm remaining mostly intact. This may be because the tortuous and narrow grain boundary path hindered the transport of the molten salt and specifically the inward diffusion of the corrosive impurities or the outward diffusion of the corrosion products, thereby slowing down the overall grain boundary corrosion.

## Conclusion

In this work, molten salt dealloying was applied to bulk foils to study the morphological and elemental evolution when forming a porous structure. The pore formation of Ni–20Cr foils was investigated in three molten salt conditions: KCl–MgCl_2_ in vacuum, and KCl–NaCl in vacuum and in air, for reaction times of 1–16 h at 700 °C. The selective corrosion formed micro-pores and ligaments on the surface of Ni–20Cr foils while the corrosion depth was identified to be ~ 1–2 µm. The results showed that molten salt dealloying may be more suitable to fabricate porous structures in thin samples such as films or micro-particles. Future studies will investigate if the porous structure can progress deeply into a bulk sample in molten salt dealloying with a longer corrosion time, or if the corrosion process may be prohibited by a slowing down of long-range diffusion. The EDX analysis revealed that Cr leaching is the main reaction during the process. The oxide regions were also determined to contain higher Cr and O contents. This oxide was identified as a Cr_2_O_3_ phase by XRD in a reflection geometry. During corrosion in KCl–MgCl_2_ under vacuum, the feature size increased drastically due to coarsening. In contrast, the corrosion behavior in the other two conditions was not affected by coarsening with relatively small feature size, likely due to the presence of a higher oxide content because of the use of the lower-purity NaCl. This work expanded our fundamental understanding of MSD and further explored the feasibility of using MSD to fabricate porous structure in different salt systems. Future work may continue exploring MSD under the influence of different alloy composition, molten salt species and impurities to establish quantitative measurement to control the porosity, pore size and ligament size for creating functional porous materials.

## Supplementary Information

Supplementary Video 1.

Supplementary Information 1.

## Data Availability

The datasets used and/or analyzed during the current study are available from the corresponding author upon reasonable request.
