# Supplementary material for: Evolution of micro-pores in Ni–Cr alloys via molten salt dealloying
Source: Sci Rep. 2022 Dec 1;12:20785. doi: 10.1038/s41598-022-20286-5 (PMC9715680; doi:10.1038/s41598-022-20286-5)
Supplement: Supplementary file 2 — Supplementary Information 1. [file 41598_2022_20286_MOESM2_ESM.docx]

**Supporting Information**


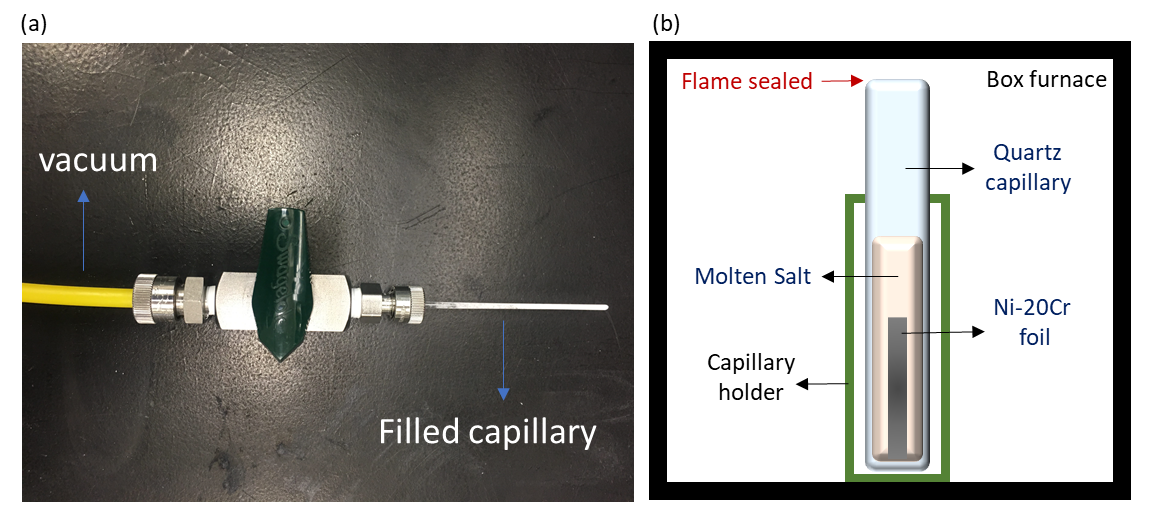


Figure S1 (a) photograph of the vacuum adapter built in-house. (b) A schematic showing the corrosion experimental set up.


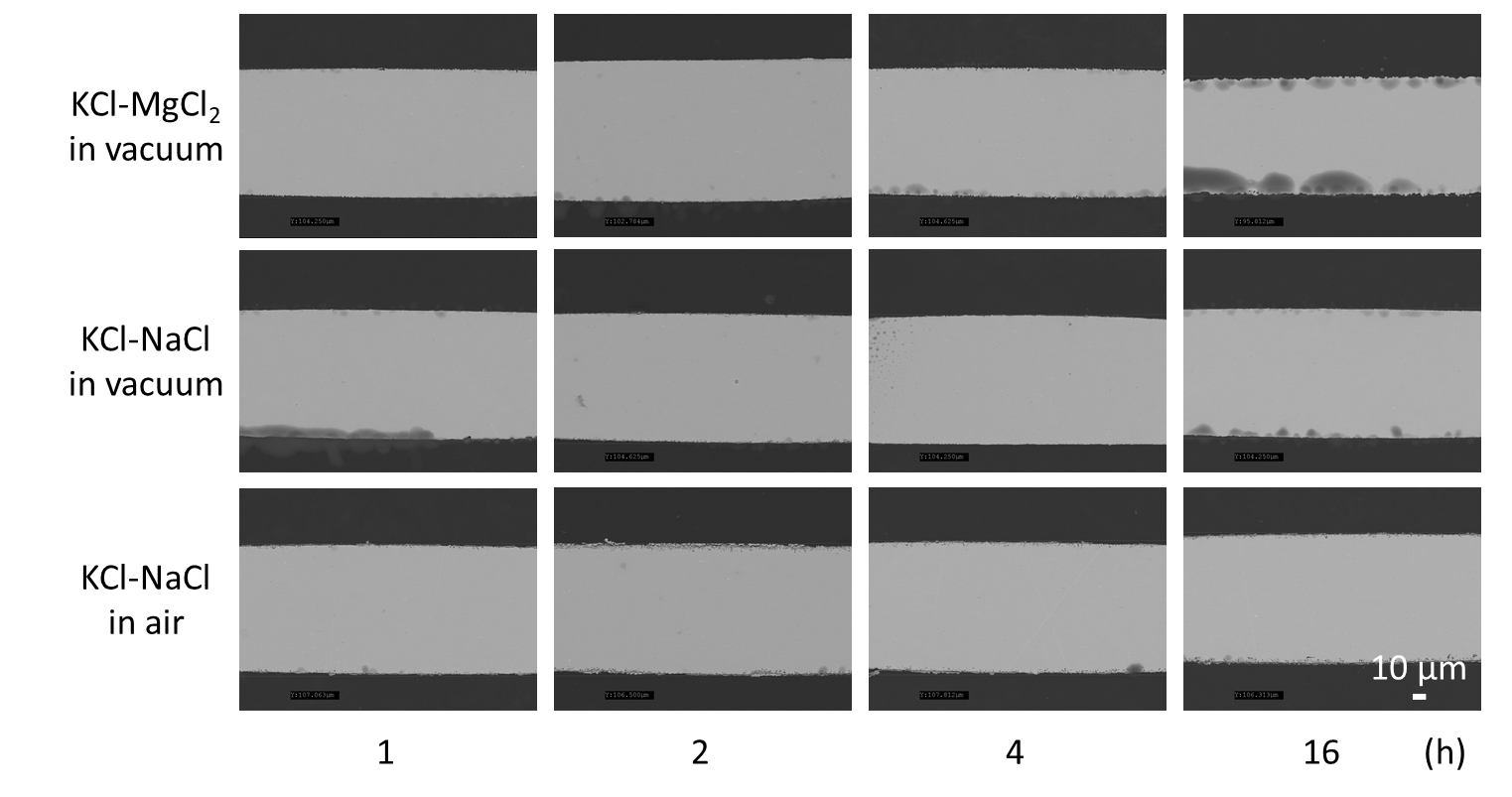


Figure S2 The SEM images of Ni-20Cr corrosion in molten salts under different conditions for thickness measurement.


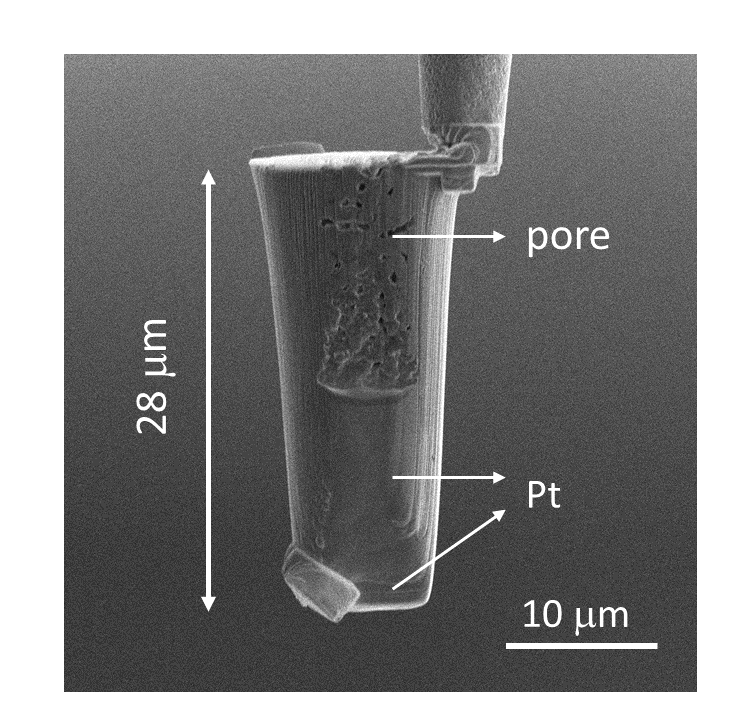


Figure S3 The SEM image of Ni-20Cr sample corroded in KCl-NaCl in air for 16 h and prepared by FIB-SEM lift-out for X-ray nano-tomography studies. The height is 28 μm and the diameter is 9-10 μm. The surface is covered with Pt protection layer. Pores due to molten salt corrosion is observed on the surface.

**The ligament sizes of Ni-20Cr foil after molten salt dealloying in different conditions measured by SEM images.**


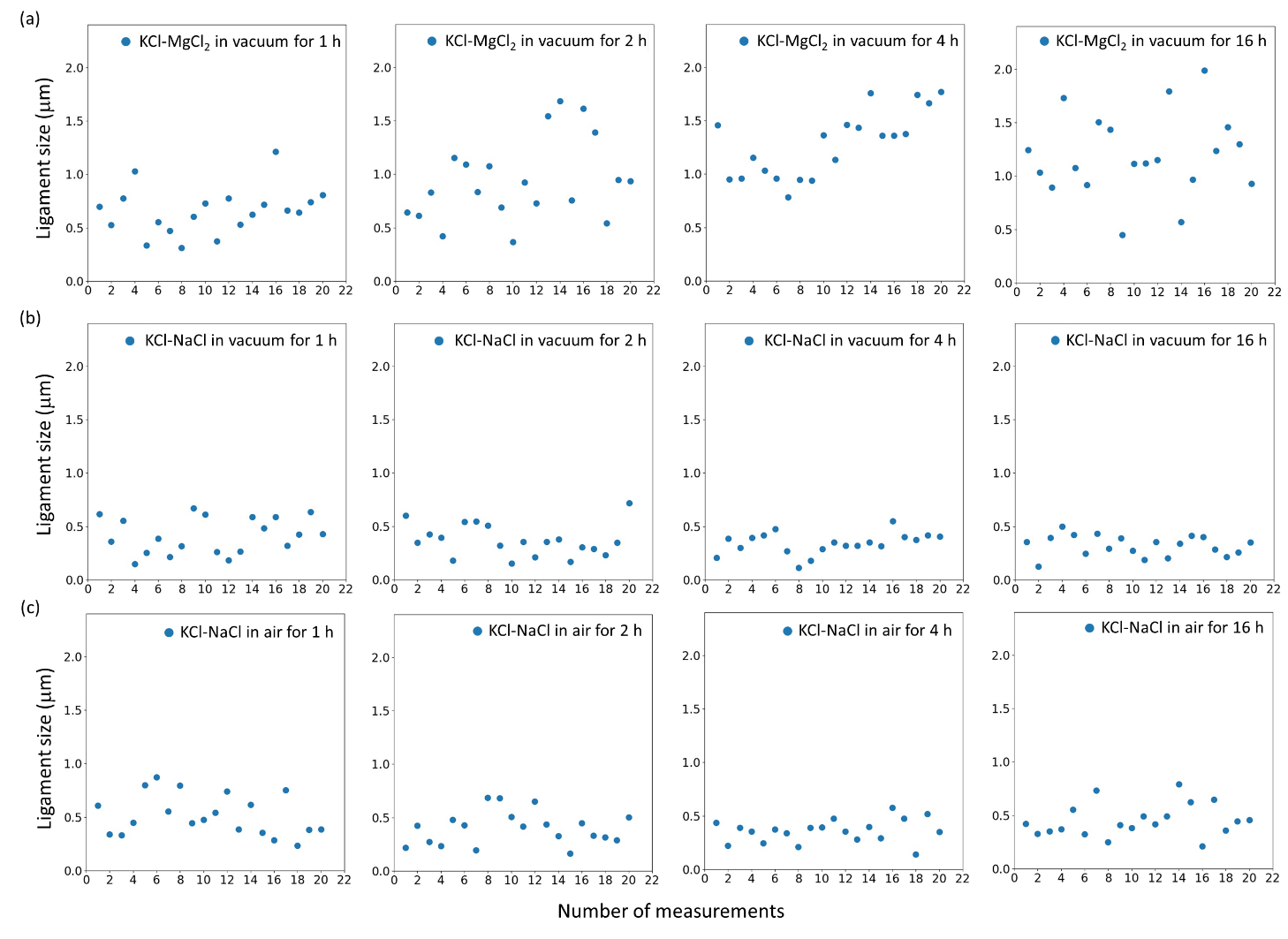


Figure S4. The quantification of the ligament sizes from 20 measurements in SEM images for the Ni-20Cr foil treated in (a) KCl-MgCl_2_ in vacuum for 1, 2, 4, 16 h; (b) KCl-NaCl in vacuum for 1, 2, 4, 16 h and (c) KCl-NaCl in air for 1, 2, 4, 16 h.

**Movie S1.** In separate file: 360° rotational view of 3D volume rendering of synchrotron X-ray nano-tomography on Ni-20Cr after corrosion in KCl-NaCl in air for 16 h. Near-surface region is shown displaying pore formation.
